# *Pedixplorer*: a Bioconductor package to streamline pedigree design and visualization

**DOI:** 10.1093/bioinformatics/btaf329

**Published:** 2025-06-03

**Authors:** Louis Le Nézet, Jason Sinnwell, Anna Letko, Catherine André, Pascale Quignon

**Affiliations:** Univ Rennes, CNRS, IGDR (Institut de Génétique et Développement de Rennes) - UMR6290, Rennes 35000, France; Department of Quantitative Health Sciences, Mayo Clinic, Rochester, MN 55905, United States; Institute of Genetics, Vetsuisse Faculty, University of Bern, Bern 3012, Switzerland; Univ Rennes, CNRS, IGDR (Institut de Génétique et Développement de Rennes) - UMR6290, Rennes 35000, France; Univ Rennes, CNRS, IGDR (Institut de Génétique et Développement de Rennes) - UMR6290, Rennes 35000, France

## Abstract

**Motivation:**

Understanding kinship relationships is fundamental to genetic research, particularly in the context of genetic linkage studies and population genetics. Pedigree design and analysis are a prerequisite for these investigations. The legacy kinship2 CRAN package has been a cornerstone in this area; however, the need for handling larger and more complex datasets necessitates an updated, flexible, and user-friendly toolset. To address this issue, we present Pedixplorer, a novel Bioconductor package designed to enhance kinship analyses with modern functionality and usability, especially in large multigeneration complex pedigrees with inbreeding loops, which are frequently seen in domestic animal breeding.

**Results:**

Pedixplorer builds upon the robust foundation of kinship2, integrating Bioconductor standards and most recent programming practices. Its core component is the S4 Pedigree object, facilitating efficient representation of complex pedigrees. The new functions enable automatic querying, filtering, and trimming of large pedigrees, while the graphical functions have been rewritten for better customization in pedigree visualizations. Additionally, Pedixplorer offers a comprehensive Shiny application, accessible both locally and via a dedicated website, allowing non-R users to easily create, filter, and customize pedigrees.

**Availability and implementation:**

The Pedixplorer package is freely available at: https://www.bioconductor.org/packages/release/bioc/html/Pedixplorer.html with additional documentation at https://louislenezet.github.io/Pedixplorer. A user-friendly web application is available at: https://pedixplorer.univ-rennes.fr.

## 1 Introduction

Pedigree analysis has historically been a cornerstone of genetic research, offering critical insights into inheritance patterns, familial aggregation of diseases, and population genetics. While the rise of high-throughput sequencing technologies and genome-wide association studies (GWAS) has led to a decline in traditional pedigree-based studies, linkage analysis remains highly relevant (Ott *et al.* 2015). It offers a powerful approach for detecting genetic loci with strong effects in studies with small sample sizes. Furthermore, it facilitates the discovery of rare genetic variants inherited within families (Bailey-Wilson and Wilson 2011), and novel methods are being proposed to leverage pedigree information within whole-genome sequencing data (Ott *et al.* 2015). Large-scale pedigree analysis is also essential in studies of isolated human populations, such as those conducted in Quebec, where extensive and complex family structures are used to elucidate genetic structure (Roy-Gagnon *et al.* 2011). In addition, pedigree analysis allows for better management of animal breeding practices, resulting in healthier animal populations, and remains a fundamental component of genetic counseling in human medicine (Genetic Alliance and Collaborative The New England Public Health Genetics Education Collaborative 2010).

Pedigrees are crucial in various fields, including medical and forensic genetics, animal breeding, and ecology. Researchers in these areas need tools for visualizing complex genealogical relationships, managing large datasets, and calculating kinship coefficients.

Since the early 2000s, more than 25 software programs (e.g. Haploforge (Tekman *et al.* 2017), HaploPainter (Thiele and Nürnberg 2005), Pelican (Dudbridge *et al.* 2004)) have been created to visualize and manage pedigrees. However, most are now deprecated (see Table 1, available as supplementary data at *Bioinformatics* online for details) even if some software packages remain actively maintained. Standalone software includes Madeline2.0 PDE (Trager *et al.* 2007), Cranefoot3.2 (Mäkinen *et al.* 2005), DrawPed (Schönberger *et al.* 2024), pedigreejs (Carver *et al.* 2018), and OpenPedigree (Buske *et al.* 2025). Additionally, package-based solutions include QuickPed (Vigeland 2022), which is built on kinship2 (Sinnwell *et al.* 2014). Each program has unique features but adheres to the recommendations of the Pedigree Standardization Task Force (PSTF) (Bennett *et al.* 2008) to varying degrees. The PSTF was created to create uniform graphical and semantic conventions for pedigree representation in clinical and research settings. Their guidelines are necessary to avoid discrepancies in layout, symbol usage, or metadata that could lead to confusion and hinder collaboration.

One of the main challenges in pedigree visualizations is to properly align individuals. Existing software often struggles with intricate structures such as cross-generational matings, multiple complex and/or repeated loops, monozygotic twins, as well as large litters and extremely large pedigrees. Madeline 2.0, DrawPed, and pedigreejs struggle with cross-generational matings, and their alignment is disrupted by consanguinity. In contrast, the alignment algorithms of kinship2 and OpenPedigree remain accurate in these cases. QuickPed requires extensive manual data entry for each individual, making it impractical for large-scale pedigrees. As for OpenPedigree, it can display only one affection status at a time and does not support visualization of twins nor spouses without children. These limitations highlight the need for a more flexible and user-friendly alternative capable of accommodating these complex scenarios.

The *kinship2* package (Sinnwell *et al.* 2014) written in the R programming language (R Core Team 2025) was a significant advancement in pedigree alignment and visualization. Originally developed to complement the *coxme* package for mixed-effects Cox models (Therneau and Grambsch 2000), *kinship2* was the first Comprehensive R Archive Network (CRAN) (R Core Team 2025) package available for pedigree drawing, and is used by other packages such as *FamAgg* (Rainer *et al.* 2016), *Familias* (Simonsson and Mostad 2016), *QuickPed* (Vigeland 2022), or *GESE* (Qiao *et al.* 2017) for the pedigrees’ alignment and drawing. However, its maintenance has ceased, and the landscape of genetic epidemiology demands a modern, robust, and scalable solution to meet the needs of complex, large-scale pedigree studies.

To address this gap, we introduce *Pedixplorer*, an up-to-date R package designed for handling complex pedigrees, released on the open-source Bioconductor platform (Gentleman *et al.* 2004). R is widely used for statistical computing and bioinformatics, with CRAN serving as its primary repository for general-purpose packages, while Bioconductor specializes in bioinformatics and computational biology tools.

Pedixplorer builds upon the existing codebase of kinship2, refactoring it to work seamlessly within R’s S4 object-oriented framework and aligning it with current Bioconductor standards. It provides a comprehensive suite of tools for pedigree creation, validation, visualization, and analysis, ensuring compatibility with Bioconductor’s bioinformatics ecosystem. Moreover, Pedixplorer adheres to Findable, Accessible, Interoperable, and Reusable (FAIR) data principles, promoting reproducibility and accessibility in genetic research.

In addition to all the previous functions available in *kinship2*, *Pedixplorer* offers several new features tailored to current genetic research:

- Enhanced data normalization and validation workflows to ensure pedigree integrity.
- Comprehensive pedigree visualization with customizable parameters.
- Informative metrics from proband selection to automatically refine large pedigrees.
- Comprehensive Shiny (Chang *et al.* 2024) web application for inexperienced R users.

In this article, we outline *Pedixplorer*’s core functionalities, its applications in genetic research and breeding problematics, and discuss the ways in which it addresses the limitations of existing tools. Pedixplorer aims to streamline pedigree-based workflows and facilitate new discoveries in genetic epidemiology, population genetics, and molecular genetics by empowering users with an open and flexible solution.

## 2 Results

### 2.1 Enhanced visualization and customization

The main graphical functions have been rewritten and restructured into a two-step process:

1. Data preparation: Converts a Pedigree object into a structured data frame containing all graphical elements needed for plotting.
2. Plot generation: Uses the prepared data frame, along with user-defined settings, to render the pedigree visualization.

This modular approach simplifies debugging by allowing users to inspect and modify the intermediate data frame prior to rendering the final plot, making it easier to identify and resolve issues in the visualization process. Furthermore, it enhances flexibility for advanced users, who can fine-tune the pedigree representation by adjusting colours, text sizes, or repositioning labels to highlight specific individuals or improve readability according to their needs.

Additional enhancements include:

- Gradient colouring: using the ‘generate_colors()’ function allows the user to specify the continuous trait of an affection to accurately represent quantitative data by colour gradients.
- Interactive plots: the pedigree can additionally be drawn using the *ggplot2* package (Wickham 2016). Using the *plotly* package (Sievert 2020) allows zooming and displaying information by hovering over specific individuals.
- Improved support for complex layouts: in complex pedigrees where individuals span multiple generations, the tool can now bypass spouse alignment to allow the pedigree rendering. A comparative example is provided in Fig. 2, available as supplementary data at *Bioinformatics* online.

To contextualize Pedixplorer’s strengths, we compared its features with those of existing pedigree visualization tools (see Table 3, available as supplementary data at *Bioinformatics* online). While several tools support basic pedigree drawing, Pedixplorer is the only one able to support the 17 listed features of the PSTF. Notably, only 6 out of 16 tools support multiple affection status visualization, and none offer gradient-based coloring, a key feature for representing quantitative traits or risk scores.

### 2.2 *Shiny* web application

To facilitate usage for non-programmers, Pedixplorer includes a *Shiny* (Chang *et al.* 2024) application accessible via ‘ped_shiny()’ or via a dedicated web server hosted by the University of Rennes (https://pedixplorer.univ-rennes.fr/). This application has a user-friendly design and visualization of the pedigrees. The application is built using a modular design where each component operates as a stand-alone Shiny module, communicating with other modules through reactive objects. This modular approach provides several key advantages: it simplifies maintenance, promotes code reusability, and enables separate documentation and unit testing for each module. The main components of the application include:

- Data Upload: Handles importing pedigree datasets in various formats (e.g. csv, tsv, xlsx, and rda).
- Health and Family Selection: Allows users to select a family and to choose which health condition to use as affection status.
- Filtering: Provides dynamic filtering options to subset pedigree data.
- Plot Customization and Rendering: Enables users to fine-tune visual elements like colours and information to display upon hovering over an individual before generating the final pedigree plot.
- Data and Plot Download: Facilitates the export of processed data and the customization of plots in different formats.

This modular design approach ensures that each component can be developed, tested, and documented independently. This enhances the maintainability and adaptability of the codebase to future improvements. The use of reactive objects for communication between modules facilitates the propagation of modifications across modules while reducing the computational cost by only processing the new data when necessary. This structure also makes it easier to extend the application with new features and to integrate it with other tools, thereby providing a flexible and robust solution for pedigree design and analysis.

### 2.3 Refining of large pedigrees

Handling large pedigrees can be cumbersome, making it necessary to extract and focus on relevant subsets for clearer visualization. Pedixplorer introduces the new ‘useful_inds()’ function, which identifies informative individuals based on specified probands and a maximum kinship-based distance. The distance Di, j is derived from the kinship coefficient Ki, j using the formula:

$$Di,j=\log2(\frac{1}{Ki,j})$$

This transformation makes kinship values more intuitive by representing them as discrete steps in the pedigree. For example, siblings (with *K* = 0.25) have a distance of *D* = 2, while cousins (with *K* = 0.0625) have a distance of *D* = 4. By setting a threshold on D, users can dynamically filter pedigrees to retain only individuals closely related to the probands, effectively reducing visual complexity while preserving key familial relationships. Parents who are needed to keep the structure of the pedigree will also be kept. This metric allows researchers to focus on the most relevant portion of the pedigree without manually tracing connections. Consequently, the interpretation of large and complex family trees becomes faster, easier, and more straightforward.

### 2.4 Pedigree S4 object

One of the major improvements in the *Pedixplorer* package is the handling of pedigree data. In contrast to the *Kinship2* management of pedigree data through an S3 object, our implementation utilizes a structured S4 object. S3 objects, commonly used in base R, offer a lightweight and flexible approach, relying on generic functions and implicit class structures. However, this flexibility comes at the cost of weaker validation and less formalized data integrity, which can lead to inconsistencies and harder-to-track modifications. In contrast, the formal definition of S4 classes ensures that data structures are rigorously defined, reducing the likelihood of errors and making interactions more predictable for users. Additionally, S4 objects support inheritance, allowing other packages to extend the Pedigree class and integrate seamlessly with Pedixplorer. This design choice aligns with Bioconductor standards, promoting interoperability within the bioinformatics community and facilitating collaborative developments. Although S4 objects require more careful planning and upfront design, this investment ultimately results in code that is unambiguous, easily maintained, and consistent throughout the package.

The Pedigree object contains four slots, each storing a different S4 object for a specific type of information essential for the pedigree construction (details are available in File 4, available as supplementary data at *Bioinformatics* online).

- ‘ped’ (Ped object): Stores basic pedigree information (i.e. identity, disease, proband, other information)
- ‘rel’ (Rel object): Describes special relationships that cannot be captured in the ped slot (i.e. twins, spouse with no child)
- ‘scales’ (Scales object): Provides plotting information (i.e. filling and bordering of the symbols)
- ‘hints’ (Hints object): Provides horizontal ordering between spouses and siblings

This structure ensures data integrity by allowing validation rules and interconnection between the different objects. For example, all individuals referenced in the ‘rel’ object must exist in the ‘ped’ object, and monozygotic twins must share the same sex.

To facilitate the use of this new data structure and to ensure a smooth transition from *kinship2*, multiple constructors have been added and are interoperable with the previous kinship2 version. Standardization steps have also been incorporated to automatically detect and correct errors, such as the mis-gendering of a parent or loop detection when one individual is its own ancestor. The latter was not checked in kinship2 and the consequent errors were complicated to understand and identify.

### 2.5 Kinship matrix analysis

*Pedixplorer* also provides functionality for computing kinship matrices. The kinship function from *kinship2* based on Lange (1997) has been rewritten as an S4 generic method, allowing it to operate directly on Pedigree objects while accounting for monozygotic twins present in the Rel object. To maintain backward compatibility, it also supports input as a Ped object or a sequence of character vectors. Kinship analysis can be performed for autosomal chromosomes or restricted to sex chromosomes, providing flexibility for different genetic studies. The output is a sparse block-diagonal matrix, structured by family units to optimize storage and computation.

### 2.6 Documentation and unit-testing

All *kinship2* package documentation and vignettes have been reviewed, improved, and expanded. Function documentation has been rewritten using the *roxygen2* package (Wickham *et al.* 2024), enabling in-place documentation and simplifying updates. The revised documentation is now accessible via a dedicated website (https://louislenezet.github.io/Pedixplorer/), facilitating user navigation and retrieval of information.

Testing is now implemented using the *testthat* (Wickham 2011) and *shinytest2* packages (Schloerke 2024), achieving over 90% code coverage. This robust testing framework ensures better tracking of modifications and enhances code stability.

## 3 Discussion

We developed the *Pedixplorer* package in response to the lack of accessible, user-friendly pedigree software and the lack of maintenance of the *kinship2* package. In accordance with the Bioconductor community guidelines, *Pedixplorer* is based on the *kinship2* package, which has been thoroughly revisited and modernized using S4 objects and a Shiny application to design and visualize complex pedigrees. This architecture offers a robust foundation for future extensions that bridge pedigree visualization with genomic analysis. A natural evolution of the package would involve interoperability with tools that provide genetic data annotations, enabling users to overlay key genetic information—such as carrier status for pathogenic variants, allelic segregation patterns, or polygenic risk scores directly onto pedigree plots.

*Pedixplorer’*s intuitive interface and extensive documentation make it suitable for both novice and experienced genetic researchers. The inclusion of an R Shiny application further enhances usability by providing a graphical interface for users who may not be familiar with R programming language. This application can be accessed either locally or via a dedicated web server, thus offering flexibility in terms of usage.

By providing *Pedixplorer*, we aim to foster new collaborations. While the package has primarily been used within our genetic research teams, we invite feedback from the community to further refine and improve its use and functionality. Contributions and feedback can be shared via the dedicated GitHub repository (https://github.com/LouisLeNezet/Pedixplorer).

To ensure the long-term maintenance of Pedixplorer, the first author is committed to actively developing and updating the package, addressing issues and feature requests through the GitHub issue page. Additionally, the web server hosting the Shiny application is expected to remain operational, as there are no anticipated issues with the maintenance of the virtual machine at the University of Rennes.

## Supplementary Material

btaf329_Supplementary_Data
