# Supplementary material for: Pedixplorer: a Bioconductor package to streamline pedigree design and visualization
Source: Bioinformatics. 2025 Jun 3;41(6):btaf329. doi: 10.1093/bioinformatics/btaf329 (PMC12203550; doi:10.1093/bioinformatics/btaf329)
Supplement: btaf329_Supplementary_Data [file btaf329_supplementary_data.zip › SupplementaryS4_File_PedigreeObject.pdf]

Supplementary S4 : Pedigree object

- `ped` (Ped object): Stores basic pedigree information with the following slots:
  - Identifiers: `id`, `dadid`, `momid`, `famid` (i.e. individual, his father, mother and family)
  - Information influencing the shape of the symbols:
    - `sex` (“male”<”female”<”unknown”)
    - `miscarriage` (TOP` = Termination of Pregnancy, `SAB` = Spontaneous Abortion, `ECT` = Ectopic Pregnancy, `FALSE` = no miscarriage)
  - Information influencing the colors of the symbols:
    - `avail`: availability status of the individual (`TRUE`, `FALSE`, `NA`)
    - `affected` (`TRUE`, `FALSE`, `NA`)
  - Additional information plotted next or upon the symbols:
    - `evaluated`: is documented evaluation of the individual available (`TRUE`, `FALSE`)
    - `consultand`: is the individual seeking genetic counseling/testing (`TRUE`, `FALSE`)
    - `proband`: is the individual an affected family member coming to medical attention independent of other family members (`TRUE`, `FALSE`)
    - `carrier`: does the individual has the genetic trait but is not likely to manifest the disease regardless of inheritance pattern (`TRUE`, `FALSE`, `NA`)
    - `asymptomatic`: is the individual clinically unaffected at this time but could later exhibit symptoms (`TRUE`, `FALSE`, `NA`)
    - `fertility`: is the individual `fertile`, `infertile` or infertile by choice or unknown reason `infertile_choice_na`
    - `deceased` (`TRUE`, `FALSE`)
    - `adopted` (`TRUE`, `FALSE`)
    - `dateofbirth`
    - `dateofdeath`
- `rel` (Rel object): Describes special relationships that cannot be captured in the ped slot, with the following slots:
  - `id1`, `id2`: identifier of the two individuals
  - `code`: relationship type `MZ twin` (monozygotic twins) < `DZ twin` (dizygotic twins) < `UZ twin` (unknown zygosity twins) < `Spouse` (spouse with no child)
- `scales` (Scales object): Provides plotting information, including:
  - `fill`: data frame specifying how each status (e.g., affected) present in the Ped object should be visually represented in the pedigree. This includes colors, angles, and density patterns used for shading the individual's box.
  - `border`: data frame specifying the visual attributes of the box border (e.g., to indicate sample availability)
- `hints` (Hints object): Provides information for the alignment of the individuals:
  - `horder`: numeric vector to order individuals horizontally
  - `spouse`: matrix to horizontally order spouses
