# Supplementary material for: Pedixplorer: a Bioconductor package to streamline pedigree design and visualization
Source: Bioinformatics. 2025 Jun 3;41(6):btaf329. doi: 10.1093/bioinformatics/btaf329 (PMC12203550; doi:10.1093/bioinformatics/btaf329)
Supplement: btaf329_Supplementary_Data [file btaf329_supplementary_data.zip › SupplementaryS2_Figure_ComplexPedigreeExample.pdf]

Supplementary S2 : Multi-marriage and generational spanning complex pedigree

Ped file used:

A	A	0	0	1
A	B	0	0	2
A	C	0	0	1
A	D	A	B	2
A	E	C	B	2
A	F	C	B	1
A	G	A	E	2
A	H	F	G	1

Pedixplorer:

```
# First pedigree with no reordering
> pedi <- plink_to_pedigree("test.ped")
> plot(pedi, align_parents = FALSE, title = "1) Without reordering")

# Second pedigree with G and F spouse reordered
> hints(pedi) <- auto_hint(pedi, align_parents = FALSE)
> spouse(hints(pedi)) <- data.frame(idl = "G", idr = "F", anchor = "left")
> plot(pedi, align_parents = FALSE, title = "2) With ordering")
```

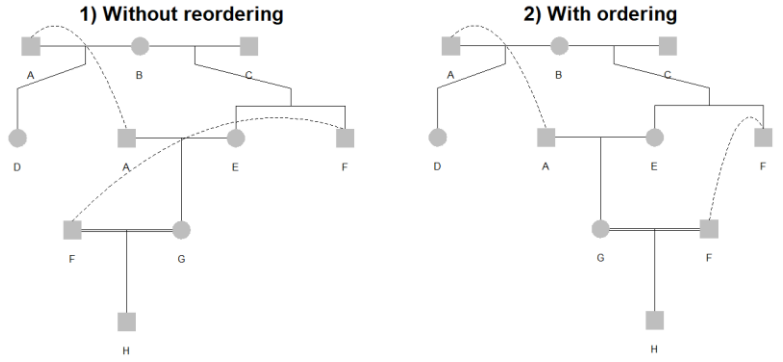

Madeline2.0

Madeline web interface is no more working. The local installation was complex but did work on linux.

```
> madeline2 test2.data
```

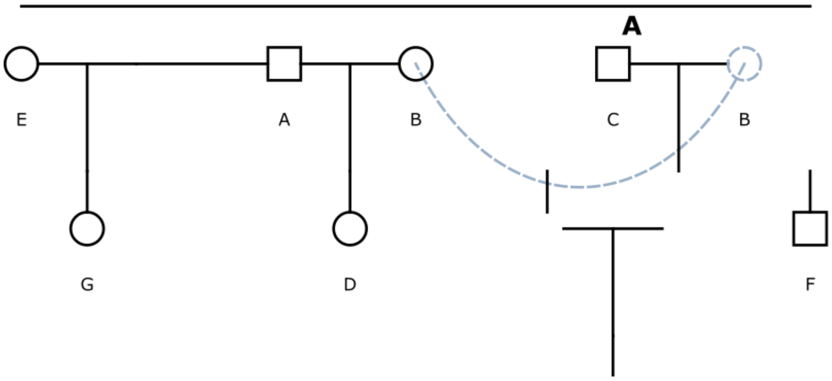

Peddraw

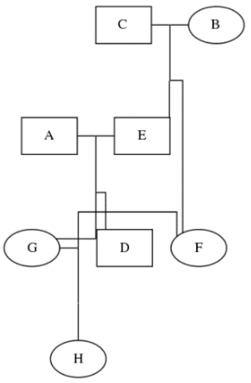

Other tested software

- **DrawPed** throw “error 64”.
- **QuickPed** and **pedigreejs** doesn’t allow to use the same individual in multiple relationship. Therefore this pedigree cannot be plot.
